# Increased Diagnostic Yield of Tuberculous Serositis by Using Serous Fluid Drainage Flocky Precipitate (SFDFP) as a Testing Sample

**DOI:** 10.1038/s41598-018-35942-y

**Published:** 2019-02-06

**Authors:** Xuhui Liu, Lu Xia, Aimei Zhang, Yao Zhang, Yuhong Liu, Shuihua Lu, Yuanlin Song, Shanqun Li

**Affiliations:** 1 Shanghai Public Health Clinical Center affiliated to Fudan University, Shanghai, China; 2 China Tuberculosis Clinical Trial Consortium, Shanghai, China; 3 Shanghai Zhongshan Hospital affiliated to Fudan University, Shanghai, China

## Abstract

A definitive diagnosis of tuberculosis serositis (TS) is still challenging. Our preliminary practice found that Serous Fluid Drainage Flocky Precipitate (SFDFP) was a useful testing sample to diagnose TS. We designed this study to assess the diagnostic performance of SFDPF for TS compared with conventional bacteriology methods on serous fluid (SF). A cohort study was conducted from July 2014 to April 2016. Patients with suspected TS were consecutively screened. SF and SFDFP were collected and tested by Ziehl-Neelsen stain, MTB culture, and Xpert/RIF assay. We compared the diagnostic performance of SF and SFDFP in several test settings. Through this study, 85 patients were enrolled, of whom 70 (82.4%) were confirmed TS or highly probable TS, 13 (15.3%) were none-TS and 2 (2.4%) indeterminate results were ruled out. The overall sensitivity using both SFDFP and SF was significantly higher than each (60% vs. 48% and 41%, p < 0.05). SFDFP and SF samples had similar diagnostic performance (p < 0.05). No false positive was detected in this study. We concluded that SFDFP is a reliable testing sample for diagnosing tuberculous serositis. SFDFP may significantly improve the diagnostic yield as a supplement to conventional tests.

## Introduction

According to the latest WHO tuberculosis report^1^, TB remains a major public health problem globally. Extrapulmonary tuberculosis contributed to 9.2% to 11.2% of all active tuberculosis in China^2^. Tuberculous serositis (TS), which includes tuberculous pleuritis, peritonitis and pericarditis, is a common form of extrapulmonary tuberculosis. A definitive diagnosis of TS to exclude effusions with other etiologies is always challenging. Culture of the bacteria from serous effusion or tissue biopsy specimens is considered to be the gold standard for the diagnosis of TS. However, culture of MTB from effusions is very time consuming and the sensitivity is not good enough. Thoracoscopy biopsy is considered to be a most accurate procedure, but it is an invasive technique associated with major (1.8%) and minor (7.3%) complications which renders this diagnostic method unsuitable for routine practice^3–6^. Previous studies reported that the sensitivity of pleural effusion culture from serious fluid (SF) was 23–58% and the sensitivity of AFB smear was only 0–10%^7^. Nucleic Acid Amplification Techniques (NAAT) have also been considered to be reliable for diagnosing pulmonary tuberculosis. But current data showed unsatisfactory diagnostic performance of NAAT for TS^7,8^. In a systematic review, the sensitivity of Xpert/RIF Assay (an innovative NAAT approved by WHO) with pleural effusion samples was reported to be 34%^8^.

**Figure 1:**
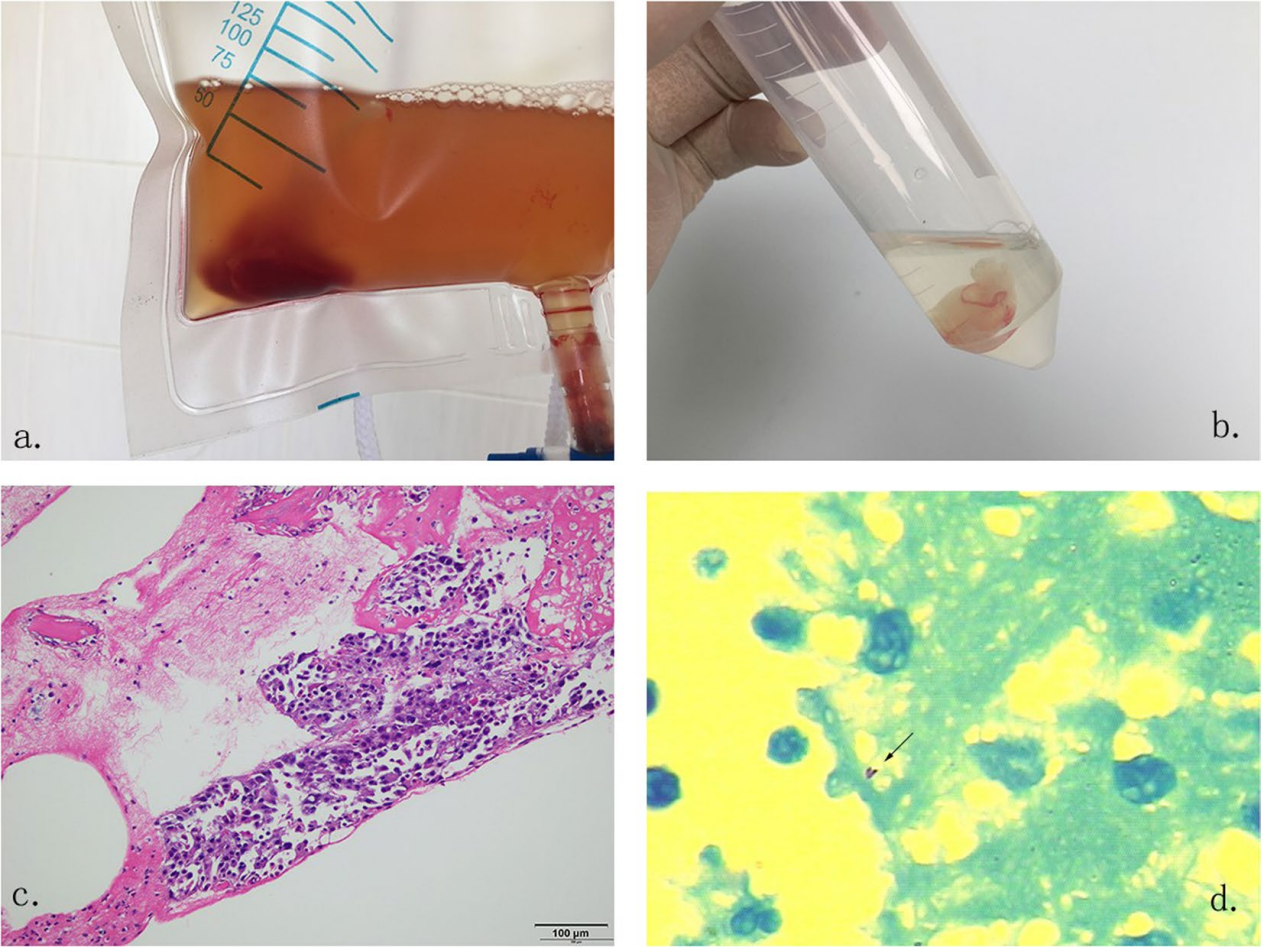
Serous Fluid Drainage Flocky Precipitate samples extracted from serous fluid. This sample can be easily collected from an overnight drainage bag and has a unique cotton-like formation (**a**,**b**). Samples can be stained and examined by histopathological methods to differentiate malignant tissue (**c**) and MTB infection (**d**). Staining methods of c and d were hematoxylin and eosin (HE) and acid-fast staining respectively.

Serous Fluid Drainage Flocky Precipitate (SFDFP) is comprised of aggregations of fibrin in serous fluid, that absorb white blood cells, isolated tumor cells, necrotic tissues and bacteria. This kind of precipitate, especially when extracted from inflammatory exudates, is a suitable sample for laboratory testing and can be collected by overnight drainage (Fig. 1a,b). By histopathological examination, using either acid-fast staining or NAAT, we may directly identify the pathogen in effusions using SFDFP as a test sample (Fig. 1c,d). Until now, there have been limited reports about the value of SFDFP for diagnosing TS. We designed a clinical evaluation of this specimen.

## Methods

### Study design

An observational cohort study was conducted from July 2014 to December 2016 in Shanghai Public Health Clinical Center, one of the three tertiary hospitals that admit TB patients in Shanghai, China. All patients admitted into this hospital who had clinical manifestations of serositis effusion were consecutively screened and enrolled into the study if serous cavity fluid (pleural effusion, peritoneal effusion or pericardial effusion) had been properly collected. Patients were evaluated with routine diagnostic workup according to their presentations. Clinical data were extracted from patients’ medical records. For patients whose diagnosis was not established during hospitalization, a telephone interview was conducted 3–6 months later to obtain the diagnosis. At the end of follow-up, each case was classified into one of several predefined clinical categories, including confirmed TS, highly probable TS, clinically indeterminate and non-tuberculous serositis, based on the clinical, radiological, microbiological, histopathological information and response to anti-TB therapy.

### Inclusion criteria

I. Hospitalized patients with clinical manifestations of serositis effusion from whom no less than 300 ml serous cavity fluid (pleural effusion, peritoneal effusion, pericardial effusion) had been collected; II. written informed consent was obtained; III. HIV test negative.

### Exclusion criteria

I. Severe heart disease; II. severe liver or renal dysfunction; III. women during pregnancy and lactation; IV. severe mental disease; V. any other situation that was considered to be not suitable for this study.

### Categorization of the Study Population

The gold standard of TB serositis in this study was defined as “positive culture results and identification of M. tuberculosis in cavity effusion or parietal tissue specimens”^9^.

As the sensitivity of the “gold standard” was low (23–69%), we categorized the cases into different groups based on a composite standard.

I. Tuberculous serositis: (1) confirmed TB serositis: acid-fast stain or culture or nucleic acid amplification positive for MTB, OR typical histologic changes (caseous necrosis, epithelioid granuloma, etc.) AND suggestive clinical and radiological findings; (2) highly probable TB serositis, i.e. cases that could not be categorized as “confirmed TB serositis”, but the clinical manifestations, laboratory results (including adenosine-deaminase level, lymphocytosis, IGRA or TST) and radiological features were highly suggestive of tuberculous serositis AND there was an appropriate response to anti-TB therapy.

II. Non-tuberculous serositis: acid-fast stain, culture, nucleic acid amplification and histopathological examination were negative on specimens collected from sites of suspected TS AND a definite alternative diagnosis was identified after 6-month follow up.

III. Clinically indeterminate: a final diagnosis of tuberculous serositis was neither highly probable nor reliably excluded after 6-month follow up.

### Sample collection and testing

Serous effusions were collected consecutively into sterile drainage bags. SF samples were tested immediately after extraction and SFDFP samples were obtained from overnight drainage bags. All samples were processed for microbiological analysis in the Clinical Microbiology Department of Shanghai Public Health Clinical Center. Tests included Ziehl-Neelsen stain, bacterial culture (BACTEC MGIT 960 system), Xpert/RIF assay (Cepheid Inc.) and histopathology examination within 8 hours from sample collection. Ziehl-Neelsen staining was performed with a kit approved by China FDA. Bactec MGIT 960 system and Xpert/RIF assay were utilized for mycobacterium culture according to the manufacturer’s recommendations.

### Statistical and Data Analysis

The sensitivity, specificity, positive predictive value (PPV) and negative predictive value (NPV) of different testing combinations on SFDFP were analyzed to evaluate the diagnostic performance of TS compared with those of SF. Diagnostic accuracy was assessed by the area under the ROC curve. Integrated discrimination improvement (IDI) index, which is used for evaluating the added predictive ability of a new marker and assessing the diagnostic potential of candidate tests or markers was calculated to describe the added predictive ability obtained via extra SFDFP testing^10–12^. The difference in means was calculated using the Student’s t-test. Statistical significance between categorical variables was compared by Pearson’s Chi-square test. P < 0.05 was considered for statistically significant. 95% confidence intervals (95%CI) were estimated for binomial distributions. Kappa value was assessed to compare the agreement between categorical variables. Statistical analysis was performed by IBM SPSS Statistics version 22 and GraphPad Prism version 5.

### Ethics statement

This study was approved by the Ethics Committee of Shanghai Public Health Clinical Center. Written informed consent was obtained from all patients enrolled in this study.

## Results

### Demographic and clinical characteristics

**Figure 2:**
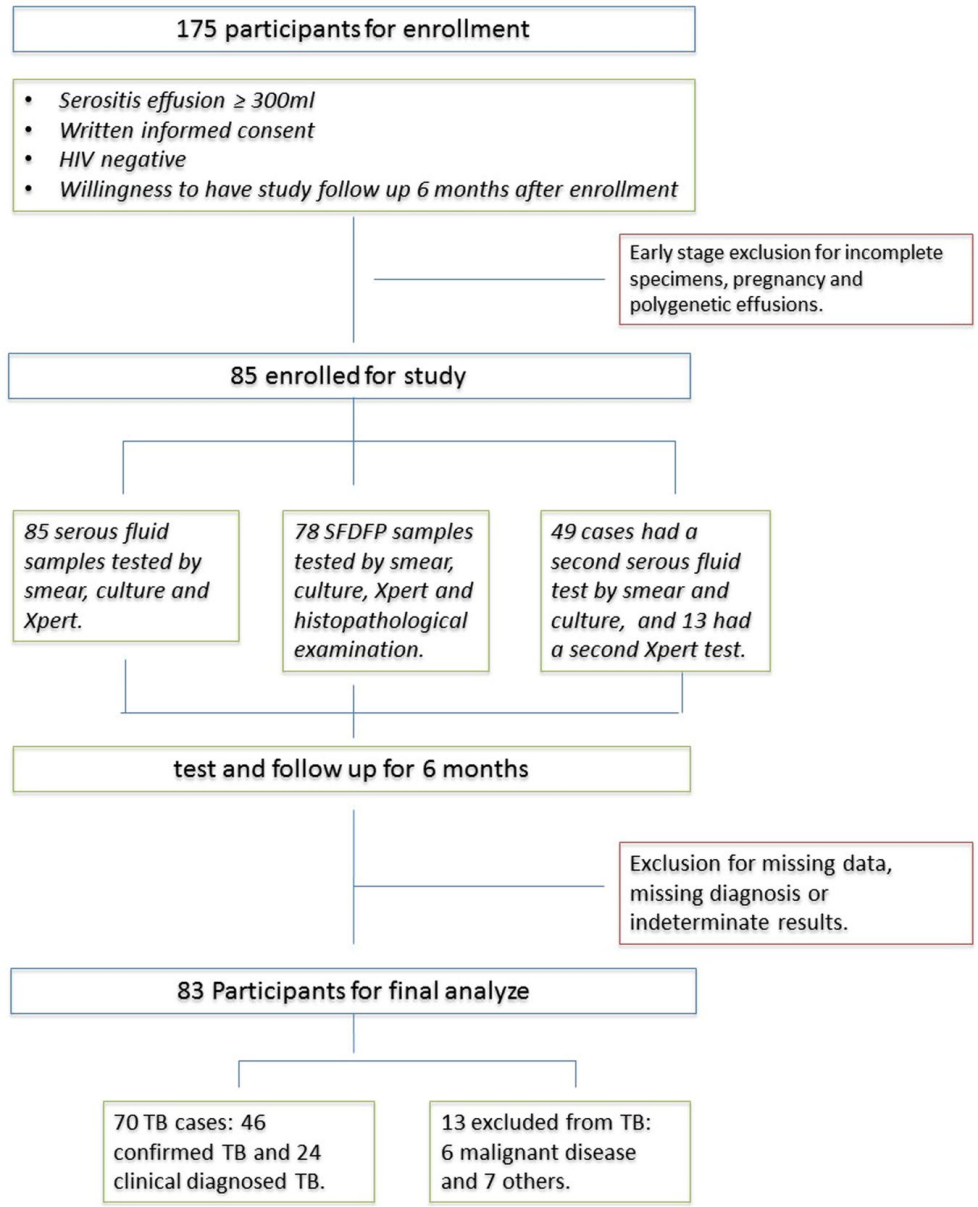
Specimen testing, participant flow and basic characteristics, enrollment and exclusion from analysis eligibility. Participants were consecutively recruited and screened at inpatient facilities in Shanghai Public Health Clinical Center with: (i) clinical suspiciion of tuberculous serositis; (ii) serous effusion over 300 ml and well collected; (iii) HIV test negative; and (iv) written informed consent obtained. The 13 second Xpert testing results were extracted from clinical data retrospectively to assess the value of the repeated test. The results of the repeated test were not included in final analysis.

From July 2014 to December 2016, a total of 175 HIV-negative patients with clinical manifestations of serositis effusion were hospitalized in Shanghai Public Health Clinical Center. Of the 175 patients, 70 patients were ruled out as follows: 2 HIV-positive, 14 refused the invasive procedure, 4 pregnancy, 37 insufficient effusion, and 13 polygenetic effusion (with severe complications, such as liver failure). Thus, 85 patients met the inclusion criteria and involved in this study. Of the 85 patients, 70 (70/85, 82.4%) were finally diagnosed with confirmed TS or highly probable TS and 13 (13/85, 15.3%) were none-TS. 2 indeterminate results were ruled out. Therefore, results from 83 patients were finally analyzed in this study. Figure 2. describes the study flow. The mean age (±SD) was 44 ± 23 years ranging from 2 to 84 years and 69.9% were male. Approximately 1/3 patients had received prophylactic or diagnostic anti-tuberculosis treatment for at least one week. For each group, TS and none-TS, the mean age, male sex and the proportion with an anti-TB therapy history were 42 ± 23 and 56 ± 19; 70.0% and 69.2%; 33.3% and 30.8%, respectively.

### Performance of different testing methods on the two specimens

The samples from the 83 patients were 67 pleural effusions and 16 peritoneal effusions (1 pericardial effusion and 1 pleural effusion were ruled out through indeterminate results). SF was obtained from all patients and invalid results were 2 Xpert/RIF errors and 1 culture failure. SFDFP was obtained from 91.6% (76/83) patients and invalid were 5 Xpert/RIF errors, 2 culture failures and 4 histopathology examination failure (due to insufficient sample tissue).

Taking clinical diagnosis as the reference, the sensitivity of integrated SF-based testing was similar to that of SFDFP (41% vs. 48%, p = 1.000), but the consistency between the two specimens was poor (kappa = 0.296). Tests on SFDFP identified 13 TB cases out of 41 SF test negative TB cases, thereby eliminating as false negative 31.7% of the negatives from conventional SF tests. If the SF and SFDFP test results were combined, the diagnostic yield increased to 60% (95%CI, 0.48–0.71), significantly higher than either the integrated SF-based test results (60% vs. 41%, p < 0.05, IDI = 0.19) or the SFDFP-based results (60% vs. 48%, p < 0.05).

In the clinical context of our study population, where the actual proportion of TS seen in this study based on the composite reference standard was 84.3% (70/83), the PPV and NPV from the SF, SFDFP and the combined SF plus SFDFP results were 100% (95%CI, 0.88–1.00) and 24% (95%CI, 0.15–0.37) respectively from SF; 100% (95%CI, 0.89–1.00) and 28% (95%CI, 0.17–0.43) from SFDFP; 100% (95%CI, 0.92–1.00) and 32% (95%CI, 0.20–0.47) from the combined results.

Subgroup analysis (Table 1) shows the test results of each bacteriological method. When comparing individual methods on SF and SFDFP, the performance of AFB smear, culture and Xpert/RIF on different samples was similar. The specificity and PPV of individual test methods were good enough, but the sensitivity and NPV were very poor (7–36% and 18–27%, respectively). Histopathological examination on SFDFP identified 11 out of 70 tuberculous effusions (15.7%) and 2 of 6 malignant effusions (33.3%). Figure 1(c,d) shows the appearance of SFDFP samples under histopathology examination.

**Table 1:** Diagnostic performance of different test combinatons.

	Valid %*	Sensitivity (95% CI)	Specificity (95% CI)	PPV (95% CI)	NPV (95% CI)	AUC*	Kappa*	P value*
Xpert/RIF on SF	97.6	0.28 (0.19–0.40)	1.00 (0.80–1.00)	1.00 (0.83–1.00)	0.23 (0.15–0.35)	0.650	0.11	0.008
Xpert/RIF on SFDFP	85.5	0.36 (0.25–0.48)	1.00 (0.78–1.00)	1.00 (0.85–1.00)	0.27 (0.17–0.40)	0.679	0.16	0.359
Smear on SF	100	0.07 (0.03–0.16)	1.00 (0.77–1.00)	1.00 (0.57–1.00)	0.27 (0.17–0.26)	0.536	0.02	0.000
Culture on SF	98.8	0.20 (0.12–0.31)	1.00 (0.76–1.00)	1.00 (0.78–1.00)	0.18 (0.10–0.28)	0.554	0.07	0.000
Smear on SFDFP	89.2	0.15 (0.08–0.25)	1.00 (0.76–1.00)	1.00 (0.70–1.00)	0.18 (0.11–0.30)	0.578	0.05	0.000
Culture on SFDFP	89.2	0.16 (0.09–0.27)	1.00 (0.76–1.00)	1.00 (0.72–1.00)	0.19 (0.11–0.30)	0.585	0.06	0.001
Pathology on SFDFP	86.7	0.19 (0.11–0.30)	1.00 (0.77–1.00)	1.00 (0.74–1.00)	0.21 (0.13–0.33)	0.657	0.08	0.001
S + C on SF	100	0.24 (0.16–0.35)	1.00 (0.77–1.00)	1.00 (0.82–1.00)	0.20 (0.12–0.31)	0.621	0.09	0.002
S + C on SFDFP	89.2	0.16 (0.09–0.27)	1.00 (0.76–1.00)	1.00 (0.72–1.00)	0.19 (0.11–0.30)	0.585	0.06	0.001
S + C + P on SFDFP	90.4	0.26 (0.17–0.38)	1.00 (0.77–1.00)	1.00 (0.81–1.00)	0.22 (0.13–0.34)	0.671	0.11	0.013
S + C + X on SF	100	0.41 (0.31–0.53)	1.00 (0.77–1.00)	1.00 (0.88–1.00)	0.24 (0.15–0.37)	0.707	0.18	—
S + C + P + X on SFDFP	91.6	0.48 (0.36–0.60)	1.00 (0.77–1.00)	1.00 (0.89–1.00)	0.28 (0.17–0.43)	0.764	0.24	1.000
All on SFDFP & SF	100	0.60 (0.48–0.71)	1.00 (0.77–1.00)	1.00 (0.92–1.00)	0.32 (0.20–0.47)	0.800	0.32	0.004
Elispot on PBMCs*	100	0.63 (0.51–0.73)	0.69 (0.42–0.87)	0.92 (0.80–0.97)	0.26 (0.14–0.42)	0.660	0.19	0.007

Notes: Abbreviated Words: “S” means smear; “C” means culture; “P” means histopathologic examination; “X” means Xpert assay; “SF” means serious fluid; “SFDFP” means serous fluid drainage flocky precipitate.

^*^“Valid” means the proportion of cases with available sample and results for all analyzed cases; “AUC” means the area under the receiver operating characteristic (ROC) curve. “Kappa” value was used to assess the agreement between the test (or test combination) and the composite reference standard (categorized as TB serositis or non-TB serositis). “p value” referred to the “S + C + X on SF” row, which stands for the routine decision-making tests to “confirm TB serositis”. The “p value” here was used to assess the statistical significance to our routine definitive methods. “Elispot on PBMC” means T-Spot test on blood samples.

Of the 13 patients who were excluded from TS diagnosis, 6 were diagnosed as non-specified parapneumonic effusions, 6 were malignant disease and 1 bacterial pericarditis. SFDFP was obtained from 12 (12/13, 92.3%) of these patients.

A sensitivity analysis was performed to evaluate the influence of the 2 indeterminate results. If the 2 indeterminate results were counted among TB cases, the sensitivity and specificity of testing based on SF + SFDFP were 58.3% (95CI, 0.46–0.70) and 100% (95CI, 0.75–1.00) respectively. If the 2 indeterminate results were seen as non-TB cases, the sensitivity and specificity were 60% (95CI, 0.48–0.72) and 100% (95CI, 0.78–1.00).

We tried to find out that whether the increased diagnostic yield was the same as the benefit from repeating conventional SF tests. So that we traced 49 medical records with a second SF test of bacteria smear and culture (37 TS and 12 non-TS) and 13 cases with a second Xpert test (6 TS and 7 non-TS) and compared the yield of SF + SFDFP and twice SF tests. One repeat of smear and culture on SF increased the positive yield from 20.4% (10/49) to 24.5% (12/49), p = 0.809, and the yield of SF + SFDFP from 20.4% (10/49) to 30.6% (15/49), p = 0.354. One repeat of Xpert on SF did not increase the positive yield (23%, 3/13) and SF + SFDFP showed an increase from 23% to 30.8% (4/13), p = 0.658.

## Discussion

For extra-pulmonary tuberculosis it is still difficult to establish definite bacteriology diagnosis. Newly published practice guidelines suggest using multiple diagnostic methods to confirm extra-pulmonary tuberculosis including AFB smear, strain culture, Xpert/RIF assay histopathology examination and IGRAs. Nevertheless, the evidence levels remained low to medium^7^. SFDFP is comprised of fibrin aggregates, blood cells, isolated tissue cells, necrotic tissues and bacteria, potentially providing a suitable sample for laboratory tests for TS diagnosis. Our preliminary study showed promising diagnostic outcome using SFDFP-based testing methods to diagnose TS. Therefore, we designed an observational cohort study to further evaluate the practicality of the SFDFP for this purpose. Multiple bacteriological testing methods for diagnosing TS were applied and the performance of different test combinations on samples of SF, SFDFP, and SF + SFDFP was evaluated (Table 1).

Overall, SFDFP gave results similar to SF but the agreement on different samples was poor (Kappa = 0.355). SFDFP based tests showed a higher sensitivity than SF (48% vs. 41%), but had more invalid test outcomes (8.4% vs. 0%). Compared with routine tests on SF samples (smear acid-fast staining, MTB culture and Xpert/RIF assay), the overall sensitivity on both SF and SFDFP samples was much higher (60% vs. 41%, p < 0.05). Tests on SFDFP samples showed potential supplemental value to tests on SF samples, with an integrated discrimination improvement of +0.19. Previous studies have shown sensitivities of 0–10% by smear, 23–58% by culture, and 56–62% by Xpert/RIF assays on pleural fluid; 0–5% by smear and 45–69% by culture on peritoneal fluid^7,13–20^. In this study, the sensitivity of individual testing methods ranged from 7% to 36%, which means that there were over 64% false negatives for each method. The yields of our culture and Xpert/RIF assays were much lower than previously reported. Probable reasons for this were: (1) approximately 1/3 patients had received prophylactic or diagnostic anti-tuberculosis treatment before testing so that the bacterial load had turned lower; (2) some patients hospitalized in our hospital were prescreened as “bacterial negative suspects” and transferred from general hospitals so that a certain proportion of patients with high bacterial load and patients with positive bacteriology result were “excluded” from our cohort. Hence, our pre-test positive patient yield was expectedly lower than in general hospitals, which greatly challenged the capability of identifying tuberculosis.

As the diagnostic yield of each individual test was low, repeating the tests was also a considerable strategy to improve sensitivity. We evaluated the yields of 2 SF samples and 1 SF sample plus 1 SFDFP sample with our limited case data. The results showed a slightly higher positive yield of 1 SF sample plus 1 SFDFP than 2 SF, but no statistically significant difference. We cannot conclude whether the increased diagnostic yield was the same as the benefit from repeating conventional SF tests in this study, but SFDFP-based testing showed a certain disagreement with SF-based testing (kappa = 0.296) and the combined sensitivity was significantly improved above SF testing alone. This implied that SFDFP is a useful approach to diagnose TS. Some published studies^21–23^ also confirmed that combined samples or repeated tests increased the diagnostic yield of MTB and single sample testing underestimated the presence of MTB, especially for low bacterial load samples. Although extra tests require more money and more time, a definitive diagnosis may prevent unnecessary drug toxicities, reduce overall costs, mitigate treatment failure and decrease the risk of dissemination and death. Accordingly, the extra tests are useful, acceptable and recommended.

In final clinical diagnosis, when using histopathological or bacteriological confirmation as the reference standard, the specificity of smear, culture, Xpert/RIF and histopathological examination tends to be very high. TB smear, culture and histopathological examination are viewed as conventional definitive methods. Previous studies showed that a false positive result from Xpert/RIF assay is rare and meta-analysis assessed the specificity of Xpert/RIF to be 98%. Our study performed Xpert/RIF assay on SF and SFDFP samples and the results shown are consistent with previous studies.

The predictive value is a most direct indicator of how reliable a testing result is and is closely related to the disease prevalence in the tested population and the sensitivity and specificity of the test itself. The prevalence of TS varies from area to area, so we cannot conclude the predictive value in other healthcare settings. Our study was performed in a tertiary hospital specialized in infectious disease in which the prevalence of tuberculosis is very high. The pre-test positive rate of TS was estimated to be as high as 80% but the sensitivity was not good enough (single test 7–36%; combined tests 16–60%), so the NPV of the tests was low (single test 18–27%; combined tests 19–32%). Because the specificity and prevalence were high, inevitably the PPV was also very high, but in this setting, neither of our laboratory tests was reliant enough to exclude TS without clinical analysis.

The diagnostic value of histopathological examination of the insoluble components from effusions in the diagnosis of extra-pulmonary tuberculosis has rarely been reported. Several studies have shown that the insoluble components of effusions are useful to diagnose malignant effusions when histopathological or immunohistochemical tests are performed on the sediment. The formation of SFDFP seems to appear more commonly in exudative effusions and is particularly a tendency in tuberculous effusion. In this study, histopathological examination on SFDFP has a positive rate of 15.7% (13/83) and identifies 2 out of 6 malignant effusions, implying its potential value for differential diagnosis. Figure 1(c,d) shows the histopathological manifestations of SFDFP identifying tuberculous and malignant effusion. If we perform histopathological examination on SFDFP as a supplement to SF testing, the sensitivity rises from 41% to 46%.

Our study has some limitations. In this observational study, we excluded those with immunosuppression status. We cannot predict how immunosuppression will influence the formation and the diagnostic utility of SFDFP because immune reactions play a very important role in the process. We aimed to test several kinds of effusions, but actually only pleural effusions and peritoneal effusions were analyzed in this study. Joint fluid was too little to harvest precipitate, and the pericarditis case was scarce in our study.

In conclusion, we found that SFDFP is useful to diagnose tuberculous serositis. This specimen can be collected along with serous fluid examination, help to increase the diagnostic yield without increasing false positives and give more chance to identify the MTB strains and get drug susceptibility results.
